# 14th YES Meeting: Breaking boundaries for the 14th time

**DOI:** 10.1097/j.pbj.0000000000000051

**Published:** 2019-08-23

**Authors:** Mariana da Silva Leal

**Affiliations:** Faculty of Medicine, University of Porto, Porto, Portugal

The 14th edition of the *Young European Scientist Meeting* has its main stage in Porto, Portugal. This year's conference will gather more than 500 students from biomedical areas, who will have the unique chance to present their research findings and to participate in the social programme, scientific sessions, practical workshops, and clinical competition.

The YES Meeting concept was born in 2006 as medical students felt the need to create a space where they would present their research findings and have the opportunity to contact and chat with scientists and physicians from all over the world. This networking need of Portuguese medical students led to 14th successful editions so far, promoting a conference which grows year after year, being the first Portuguese biomedical congress solely organized by students.

Throughout every edition, all YES Meeting Committees worked to shape the conference we see and know today. In 2019, the YES Meeting is happy to receive, in the humble city of Porto, Professor F. Ulrich Hartl, Lasker Award Basic Research winner in 2011, and Professor John van der Oost, Spinoza Prize winner in 2018, as well as experts on robotics, virtual surgery, mental health, melanoma, human genetics, ex-utero devices, among other interesting subjects.

Since 2017, the YES Meeting has established a prosperous partnership with the *Porto Biomedical Journal*, which launched the Scientific Competition further away. By visiting the following pages, readers may find all abstracts presented at the 14th YES Meeting, selected out of 262 valid submissions in the fields of *Neurosciences*, *Oncology* & *Molecular Biology*, *Public Health* & *Medical Informatics*, *Internal Medicine*, *Immunology* & *Physiology*, and *Surgery*, as well as articles written by renowned scientists, who attended previous editions of the YES Meeting. On behalf of the Organizing Committee, I would like to thank all the speakers who have embraced this initiative.

In conclusion, it is my pleasure to invite you all to read this issue's pages, born out a colligation between the YES Meeting and the *Porto Biomedical Journal*, to break boundaries one more time.

